# Primary Lateral Sclerosis: Can Rocuronium Be an Option?

**DOI:** 10.7759/cureus.35773

**Published:** 2023-03-05

**Authors:** Antonio Palha Ribeiro, Ana Sofia Tomas, Carla Oliveira

**Affiliations:** 1 Anesthesiology and Critical Care, Centro Hospitalar Universitário do Santo António, Porto, PRT

**Keywords:** sugammadex dosing and timing, non-depolarizing neuromuscular blocking agents, motor neuron disease, rocuronium, primary lateral sclerosis

## Abstract

Primary lateral sclerosis (PLS) is a neurodegenerative motor neuron disorder that is characterized by corticospinal and corticobulbar dysfunction. In this disease, muscle relaxants in general anesthesia should be used with extreme caution.

A 67-year-old woman with a history of PLS was scheduled for laparoscopic gastrostomy due to long-term dysphagia. In the preoperative assessment, she presented a tetrapyramidal syndrome with generalized muscle weakness. A priming dose of 5 mg of rocuronium was administered and the train-of-four (TOF) ratio (T4/T1) after 60 seconds was 70% so induction was followed with fentanyl, propofol, and additional 40 mg of rocuronium. After 90 seconds when T1 was lost, the patient was intubated. During surgery, the TOF ratio increased progressively until 65%, 22 minutes after a final bolus of 10 mg of rocuronium. Prior to emergence, 150 mg of sugammadex was given and neuromuscular block reversal was evidenced with a TOF ratio > 90%.

As it was decided to perform the surgery laparoscopically, general anesthesia with a neuromuscular blockade was necessary. Since it is reported that patients with motor neuron diseases show an increased sensibility to non-depolarizing muscle relaxants (NDMR), these agents should be used cautiously. Adversely to what studies document, no augmented responsiveness was shown in TOF monitoring, so the standard dose of 0.6 mg/kg of rocuronium was safely given. A final bolus of NDMR was administered after 54 minutes, demonstrating a similar pharmacokinetics profile in terms of duration of action as reported in several studies (45-70 minutes). In addition, a full and rapid neuromuscular blockade recovery with 2 mg/kg of sugammadex was seen, as previously demonstrated in a case series.

## Introduction

Primary lateral sclerosis (PLS) is an extremely rare (incidence of 1 case in 10 million), idiopathic, nonfamilial, neurodegenerative disorder with insidious onset usually in the fifth or sixth decade that is characterized by upper motor neuron dysfunction, therefore being classified as a motor neuron disorder [1].

Motor neuron disorders are a spectrum of diseases that include amyotrophic lateral sclerosis (ALS), which is the most common motor disease. Although there is much controversy about whether PLS and ALS are distinct pathological entities or not, the absence of clinical features of lower motor neuron involvement in PLS confirmed by neurophysiological studies can differentiate these disorders [2].

PLS is typically slowly progressive with an average symptom duration described in several series ranging from seven to 27 years in contrast to ALS, which has a rapidly progressive course and an average life expectancy of three years. The clinical hallmarks in PLS are restricted to corticospinal and corticobulbar dysfunction so at its onset, patients can present stiffness, clumsiness, or mild weakness with signs of spasticity and hyperreflexia, dysarthria, dysphagia, and emotional lability, while limb wasting is a rare feature of PLS compared to ALS [3]. Moreover, bladder instability resulting in frequency and varying degrees of retention is a common feature that can be observed in up to half of the patients. Even though cognitive dysfunction is generally reported as being unaffected in PLS, some case reports have documented impairment within the spectrum of frontotemporal dementia [4].

As PLS represents 1-3% of all the patients with motor neuron disease, literature concerning anesthetic management in PLS patients is scarce, with no case reports published until the date of usage of neuromuscular block agents in general anesthesia in this specific disorder. Therefore, we present the first case of balanced general anesthesia in a patient with PLS undergoing laparoscopic permanent gastrostomy and discuss perioperative concerns to consider when managing a patient with this uncommon motor neuron disease.

## Case presentation

A 67-year-old woman with a history of PLS was scheduled for laparoscopic gastrostomy due to long-term dysphagia. The patient’s PLS symptoms began 11 years before surgery when the patient presented equilibrium disturbances that resulted in frequent falls. Through the years, she developed a tetrapyramidal syndrome with generalized muscle weakness, bilateral lower extremity spasticity, and hyperreflexia as well as dysarthria and dysphagia to solids and liquids.

The patient’s surgical history was significant for thyroidectomy in 2004 and gastric bypass surgery in 2006, under general anesthesia without apparent anesthetic complications. The patient did not document any drug allergies, history of smoking, or alcohol consumption. The patient's medical history included parkinsonian syndrome, hypothyroidism, well-controlled arterial hypertension, urinary incontinence, and vitamin D insufficiency. The patient’s medication included baclofen, riluzole, levodopa, lansoprazole, levothyroxine, indapamide, oxybutynin, clonazepam, fluoxetine, benserazide, cholecalciferol, and calcium carbonate.

The preoperative assessment of the patient was done the day before the surgery where it was documented that her height was 164 cm, weight was 75 kg with a BMI of 27.89 kg/m^2^. Hemodynamically, the patient was stable with tensional values of 109/60 mmHg and other vital signs in the normal range - heart rate 65 bpm, oxygen saturation 96% at room air, and tympanic temperature 36.6ºC. The patient revealed bilateral lower extremity spasticity as well as bulbar symptoms. She presented limited functional capacity (<4 METS) due to her neurological disorder requiring wheelchair use and admitted snoring with morning sleepiness and headaches, although polysomnography did not document obstructive sleep apnea criteria. Functional pulmonary tests showed forced vital capacity (FVC), forced expiratory volume in 1s (FEV1), and ratio FEV1/FVC within the normal range. However, maximal static respiratory pressures were decreased with maximal inspiratory pressure (MIP) at 61% of predicted and maximal expiratory pressure (MEP) at 78% of predicted, therefore the patient had started noninvasive ventilation with nasal continuous positive airway pressure (CPAP) eight months prior to surgery and cough-assist two months later to facilitate mobilization of airway secretions.

**Figure 1:**
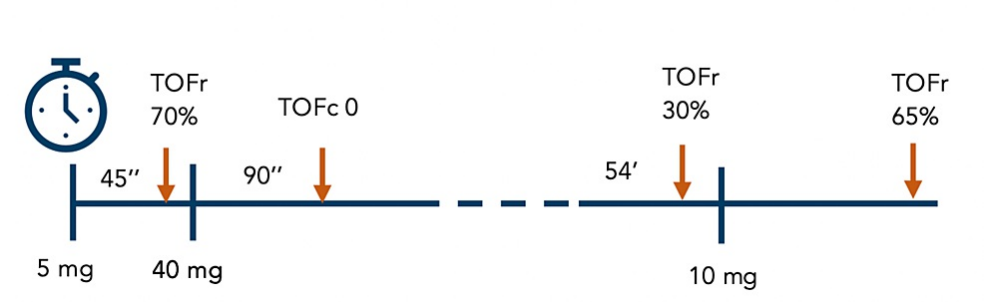
Timeline of rocuronium administrations according to quantitative neuromuscular monitoring

On the day of the surgery, the patient was premedicated with pantoprazole 40 mg and was consequently monitored following American Society of Anesthesiologists (ASA) standards (EKG, pulse oximetry, and noninvasive blood pressure device) and the bispectral index (BIS) and neurostimulator. Arterial cannulation was performed in the left radial artery in order to take serial arterial blood gas tests to evaluate oxygenation status after surgery. The patient was preoxygenated for three minutes and after a priming dose of 5 mg of rocuronium was administered, a set of train-of-four (TOF) stimuli was applied over the ulnar nerve and repeated every 12 seconds in order to access patient sensibility to non-depolarizing muscle relaxant (NDMR). As the TOF ratio (T4/T1) after 45 s was 70%, induction was followed by a bolus of fentanyl 50 mcg, propofol 100 mg, and rocuronium 40 mg. After additional 90 seconds when T1 was lost, the patient was intubated with a MAC3 laryngoscope blade without any difficulty and a 7.0 endotracheal tube was placed. Mechanical ventilation was started afterward in a pressure control-volume guaranteed mode, maintaining anesthesia with sevoflurane 2% in a 50% oxygen-50% air mixture. Fifty-four minutes later, an additional bolus of 10 mg of rocuronium was given as the TOF ratio (T4/T1) was 30%. The patient remained hemodynamically stable with oxygen saturation (SpO2) >99% and the TOF ratio increased progressively until 65%, 22 minutes after the previous and final bolus of rocuronium. A schematic chronological view of the rocuronium bolus administered according to quantitative neuromuscular monitoring is represented in Figure 1.

Prior to emergence, 150 mg (2 mg/kg) of sugammadex was given and neuromuscular block reversal was evidenced with a TOF ratio >90%. The patient was promptly suctioned and extubated after responding to verbal commands and being able to open her eyes spontaneously and consequently transferred to the hospital’s post-anesthetic care unit. Postoperatively, analgesia was managed with acetaminophen 1000 mg and for nausea and vomiting prophylaxis a bolus of ondansetron 4 mg was given. The patient was discharged five days later, hemodynamically stable with SpO2 >98% with nasal CPAP. 

## Discussion

PLS is an uncommon pre-junctional disorder that affects upper motor neurons that innervate the voluntary musculature and represents a challenge to the anesthesiologist, as patients have an increased risk of pulmonary perioperative complications. In this case, we present a patient with 11-year PLS evolution with spasticity, significant bulbar symptoms, and ventilatory support at home, which increases dramatically the risk of aspiration and respiratory failure especially in upper abdominal surgery [5]. Furthermore, it is known that gastric bypass surgery can also increase the possibility of this fatal event [6]. Therefore, it is of extreme importance to delineate a detailed anesthetic plan in order to not only prevent aspiration but also respiratory failure after the patient’s extubation.

The decision to perform the surgery laparoscopically permits the pneumoperitoneum to distend and separate the abdominal wall from its contents enhancing easier manipulation of the anatomical structures that were distorted after gastric bypass surgery [7]. Thus, general anesthesia with neuromuscular blockade was necessary in order to ameliorate the surgery’s performance. It is well-documented that depolarizing muscle relaxants, such as succinylcholine, should be preemptively avoided because this agent can lead to malignant hyperthermia, severe hyperkaliemia, rhabdomyolysis, and consequently to fatal arrhythmias [8]. For this reason, an NDMR - rocuronium - was the agent of choice in this surgery because of its fast onset time, intermediate clinical duration, and the possibility of neuromuscular block reversal with sugammadex. Since it was reported that patients with motor neuron diseases show an increased sensibility to NDMR, as loss of innervation leads to extra junctional and hypersensitive nicotinic acetylcholine receptors, these agents should be used cautiously [9]. Adversely to what the literature describes, no augmented responsiveness was shown in train-of-four monitoring, so a standard dose of 0.6 mg/kg of rocuronium was safely titrated in two boluses in this patient during induction. A final bolus of NDMR was administered after 54 minutes, demonstrating a similar pharmacokinetics profile in terms of duration of action as reported in several studies in healthy patients (45-70 minutes) [10]. In addition, a full and rapid neuromuscular blockage recovery with 2 mg/kg of sugammadex was seen, as previously demonstrated by Pühringer et al., without any signs of residual curarization and respiratory depression [11]. The decision to antagonize the neuromuscular blockade with sugammadex over neostigmine was supported by the fact that literature has shown a 22% reduction of residual paralysis with cyclodextrin reversal but also a significant reduction of respiratory complications, including pneumonia and respiratory failure as compared to the latter [12].

Moreover, it is important to consider that patients with this disorder are more sensitive to sedatives [13]. Hence, in this case, there was a cautious administration of short-acting fentanyl in order to avoid prolonged effects, chest wall rigidity, and the potentiation of respiratory depression during emergence. Sevoflurane was used to maintain general anesthesia because its short-acting effect prevents prolonged emergence, it improves respiratory mechanic parameters and enhances neuromuscular blockage.

Finally, in the postoperative period, close monitoring of vital signs especially oxygen saturation with a pulse oximeter is essential to detect respiratory depression. The eight-month history of non-invasive ventilation (NVI) and its restitution after surgery in this patient could also have a beneficial role in her respiratory function as it has been reported that NVI decreases the reintubation rate [14].

## Conclusions

In conclusion, anesthesiologists can encounter difficult challenges due to uncommon diseases and consequently anesthetic management should not only be addressed to the disorder specificities but also to the clinical presentation of the patient by the time of surgery, medical and surgical history, type of surgery, and more specifically in this case to the increased risk of aspiration and respiratory failure.

In this patient, general anesthesia was the clear option, and we can document that standard doses of rocuronium were successfully administered, as she did not show increased sensitivity and that the pharmacokinetics profile of rocuronium in this patient was comparable to healthy patients. Also, sugammadex was safely given to recover neuromuscular blockage in the recommended dose for the TOF ratio presented at the time of reversal.
